# Role of Phosphorylated HDAC4 in Stroke-Induced Angiogenesis

**DOI:** 10.1155/2017/2957538

**Published:** 2017-01-03

**Authors:** Juan Liu, Xiang Zhou, Qing Li, Shu-Min Zhou, Bin Hu, Guo-Wen Hu, Xin Niu, Shang-Chun Guo, Yang Wang, Zhi-Feng Deng

**Affiliations:** ^1^Department of Neurosurgery, Shanghai Jiao Tong University Affiliated Sixth People's Hospital, Shanghai, China; ^2^Graduate School of Nanchang University, Nanchang, China; ^3^Institute of Microsurgery on Extremities, Shanghai Jiao Tong University Affiliated Sixth People's Hospital, Shanghai, China

## Abstract

Acetylation or deacetylation of chromatin proteins and transcription factors is part of a complex signaling system that is involved in the control of neurological disorders. Recent studies have demonstrated that histone deacetylases (HDACs) exert protective effects in attenuating neuronal injury after ischemic insults. Class IIa HDAC4 is highly expressed in the brain, and neuronal activity depends on the nucleocytoplasmic shuttling of HDAC4. However, little is known about HDAC4 and its roles in ischemic stroke. In this study, we report that phosphorylation of HDAC4 was remarkably upregulated after stroke and blockade of HDAC4 phosphorylation with GÖ6976 repressed stroke-induced angiogenesis. Phosphorylation of HDAC4 was also increased in endothelial cells hypoxia model and suppression of HDAC4 phosphorylation inhibited the tube formation and migration of endothelial cells in vitro. Furthermore, in addition to the inhibition of angiogenesis, blockade of HDAC4 phosphorylation suppressed the expression of genes downstream of HIF-VEGF signaling in vitro and in vivo. These data indicate that phosphorylated HDAC4 may serve as an important regulator in stroke-induced angiogenesis. The protective mechanism of phosphorylated HDAC4 is associated with HIF-VEGF signaling, implicating a novel therapeutic target in stroke.

## 1. Introduction

Stroke is the second leading cause of long-term disability in high-income countries and the second leading cause of death worldwide [1]. The morbidity and mortality rates of stroke and resultant disability remain high despite the fact that marked improvements in medical and endovascular recanalization therapy have been achieved. Clinical and experimental data show that angiogenesis is activated after stroke, and higher microvessel density correlates positively with clinical prognosis [2, 3]. Hence, strategies to augment angiogenesis may facilitate the recovery of stroke.

Class IIa HDAC4 is a large protein with an extended N-terminal regulatory domain and a C-terminal tail. Previous studies have demonstrated that HDAC4 is highly expressed in the brain [4], and neuronal activity depends on the nucleocytoplasmic shuttling of HDAC4 [5]. HDAC4 deficiency mice display a progressive loss of neurons in the cerebellum, and forcing expression of HDAC4 protects neurons from cell death by inhibiting cyclin-dependent kinase 1 and cell-cycle progression [6]. A recent study found that HDAC4 regulates the survival of retinal neurons during retinal development and promotes the survival of retinal neurons in a mouse model of retinal degeneration [7]. Therefore, HDAC4 is a promising therapeutic target and shows application prospect in CNS diseases. However, whether HDAC4 is involved in the regulation of angiogenesis after cerebral ischemia remains largely unexplored.

In this study, we aimed to investigate the functional roles of HDAC4 in ischemic stroke and explore the underlying mechanism. We firstly showed that the level of phosphorylated HDAC4 was profoundly upregulated after ischemic stroke, and HDAC4 phosphorylation was required for postischemia angiogenesis, as inhibition of HDAC4 phosphorylation could result in a significant decrease in the angiogenic responses from the ischemic brain tissues. Then, we confirmed the phosphorylation phenomena and angiogenesis regulatory effects of HDAC4 in an endothelial cell hypoxia model. Moreover, we found that the blockade of HDAC4 phosphorylation remarkably decreased the expression of target genes downstream of HIF-VEGF signaling. These data suggest that the phosphorylation of HDAC4 is essential for angiogenesis after cerebral ischemia and the regulatory effect of HDAC4 on angiogenesis may be mediated by the regulation of HIF-VEGF signaling.

## 2. Materials and Methods

### 2.1. MCAO Model and Treatment Groups

Animal procedures were approved by the ethics committee of Shanghai Jiao Tong University and were in accordance with the guidelines of the US Department of Health for the use and care of laboratory animals. Transient middle cerebral artery occlusion (MCAO) in rats has been described previously [8]. Briefly, male Sprague-Dawley rats weighing 250 g to 300 g were anesthetized with 4% isoflurane in 70% N_2_O/30% O_2_ using a mask. Both the right common carotid arteries and the right external carotid artery (ECA) were isolated and occluded under a microscope. A 4-0 monofilament nylon suture with a heat-rounded tip was introduced into the incision of ECA and advanced to block the origin of the middle cerebral artery. After 120 minutes of ischemia, the suture was carefully removed to restore blood flow. While being under anesthesia, the rectal temperature was monitored and maintained at 37.0 ± 0.5°C using a thermal heating pad. No morphological or biochemical evidence of ischemic brain injury was noted in the left cortex. After MCAO, animals were randomly divided into 2 groups (*n* = 10/group): (1) MCAO + vehicle group (0.1% DMSO in 0.1 M PBS) and (2) MCAO + GÖ6976 group (Sigma, St. Louis, MO, USA, 2.50 mg/kg body weight). The rats were intraperitoneally injected with vehicle or GÖ6976 once daily and the dose was chosen as previous studies reported [9, 10]. MCAO rats were sacrificed and perfused at indicated time point, and brain tissues were collected for further processing.

### 2.2. Cell Culture

Primary rat brain microvascular endothelial cells (RBMEC) were cultured as described previously [11]. Briefly, cells from passage 6 to passage 8 were cultured with DMEM medium supplemented with 10% fetal bovine serum, 20 *μ*g/mL bFGF, and 100 *μ*L/mL heparin under standard conditions (5% CO_2_, 37°C, and 95% humidity). To create a hypoxic environment (1% O_2_), cells were placed in a Thermo incubator chamber, flushed with a mixture of 1% O_2_, 5% CO_2_, and 94% N_2_, and incubated at 37°C. For hypoxia experiments [12, 13], confluent RBMEC were starved with serum-free medium for 12 hours and then randomly assigned into 3 groups: N group: cells treated with DMSO cultured in normoxic condition (21% O2, 5% CO2); H group: cells treated with DMSO cultured in hypoxia condition; and H+G group: cells treated with GÖ6976 at a concentration of 1.5 *μ*M, and then they were cultured in hypoxia condition.

### 2.3. Quantitative Real-Time PCR (qRT-PCR)

Tissue and cellular RNA samples were directly extracted with TRIzol reagent (Invitrogen, USA). Total RNA was isolated according to the manufacturer's protocol. The integrity of RNA was quantified by a NanoDrop 2000 spectrophotometer (Thermo Scientific, USA). Samples with OD260/280 between 1.8 and 2.0 were used for further study. Quantitative real-time PCR was performed according to the manufacturer's instructions. Briefly, 1 *μ*g of RNA was transcribed using a RT-PCR kit (Takara, Japan). RT-PCR was performed on a fast real-time 7900 PCR System by using the SYBR Green Universal PCR Master Mix (Roche, Switzerland). Primers sequences used for RT-PCR were as follows: rat *β*-actin forward: TACAACCTTCTTGCAGCTCC; reverse: ATCTTCATGAGGTAGTCTGTC; rat HDAC4 forward: CACCTTCCCCATGTCAGTCC; reverse: ATGCACTCACACTTGCCACG; rat Rcan2 forward: GGGAGACGCCTACTTCATTGG; reverse: CAGCCCAGTCTCTGTCTATGCA; and rat Nur77 forward: GCGGCTTTGGTGACTGGATA; reverse: AGTGATGAGGACCAGAGCAGACA. The relative expression levels of the detected genes were normalized to the endogenous control (*β*-actin) in triplicate and calculated in 2^−ΔΔCT^ method. Each qRT-PCR was performed in triplicate for yield validation.

### 2.4. Immunofluorescence

For rat brain immunofluorescence, rats were killed 48 h after MCAO. Brain samples (MCA cortex area) were fixed with 4% paraformaldehyde and then dehydrated sequentially in 20% and 30% sucrose solutions until the brain sank. Tissues were stored at −80°C after being embedded in optimal cutting temperature (OCT) compound. Consecutive coronal sections of 8 um thickness were incubated in 1% BSA with mouse anti-PECAM-1 (1 : 50; Santa Cruz Biotechnology, Heidelberg, Germany) or anti-CD34 (1 : 200; Abcam Inc., Cambridge, MA, USA) at 4°C overnight. Each section was washed with PBS and incubated with Alexa-488-conjugated goat anti-mouse secondary antibodies (1 : 400) or Alexa-594-conjugated donkey anti-rabbit secondary antibodies (1 : 400) at room temperature for 2 hours the following day. 1% BSA was used as a control to confirm the specificity of the antibody and DAPI (1 : 30) was used to detect the nucleus. To quantify microvascular density after MCAO, images of the ischemic cortex and the contralateral cortex were acquired using a Leica fluorescence microscope, the PECAM-1 positive cells or CD34 were positive cells counted in each image and the mean of the total positive cell counting was considered as microvascular density [14, 15].

### 2.5. Western Blot Analysis

For Western blot analysis, brain tissues and cells were lysed in RIPA lysis buffer with phosphorylase inhibitor and protease inhibitor for 15 minutes on ice. After centrifugation at 14000 rpm for 15 minutes, the supernatant was collected and the protein content of the samples was determined by the Bradford method. Equal amounts of protein were loaded onto 10% SDS-PAGE gels and blotted onto PVDF membranes. Membranes were blocked with 4% nonfat milk and incubated with primary antibodies against HDAC4 (1 : 1000; Abcam, Cambridge, MA, USA), HDAC4 phosphorylated at Ser-632 (1 : 200; Santa Cruz Biotechnology, Heidelberg, Germany), HIF-*α* (1 : 200; Abcam, Cambridge, MA, USA), and VEGFa (1 : 200; Santa Cruz Biotechnology, Heidelberg, Germany) overnight. *β*-Actin (1 : 4000; Abcam, Cambridge, MA, USA) was used as a loading control. After washing three times with Tris-buffered saline, the membranes were incubated with HRP-conjugated anti-rabbit or anti-mouse secondary antibodies (1 : 2000) for 2 hours at room temperature. Specific binding was detected with enhanced chemiluminescence reagents. The blots were analyzed with ImageJ analysis software, and P-HDAC4 was normalized to total HDAC4 for comparisons.

### 2.6. Cell Proliferation Assay

Cell proliferation analysis was performed with Cell Counting Kit-8 (Dojindo, Kumamoto, Japan) according to the manual of the manufacturer. Briefly, RBMEC cells treated with or without GÖ6976 (1.5 *μ*M) were plated in 96-well plates in quintuplicate at 5 × 10^3^ cells for each well and cultured in hypoxia or normoxic condition. Cells were examined at 12 hours and at 24 hours. CCK-8 (10 *μ*L) was added to each well at different time points. After an incubation of 2 h at 37°C, absorbance was measured at 450 nm. Three independent replication experiments were performed.

### 2.7. In Vitro Tube Formation

Tube formation assay was performed to analyze the effect of phosphorylated HDAC4 on capillary network formation of endothelial cells [16, 17]. In briefly, RBMEC cells (2 × 10^4^) treated with or without GÖ6976 (1.5 *μ*M) were cultured in a 96-well plate coated with 100 *μ*L Growth Factor Reduced Matrigel (BD Matrigel) under hypoxia or normoxic condition. Tube formation was quantified after 12 hours by measuring the average total tube loops in 5 random microscopic fields with a computer-assisted microscope (Leica, Germany).

### 2.8. Scratched Wound Assay

Migration of RBMEC was detected using a “scratched wound assay.” Briefly, RBMEC (3 × 10^5^) was seeded in 12-well plate and grown to confluence. These confluent monolayers were scratched using a 200 *μ*L pipette tip. After being rinsed twice with PBS to remove the debris and replaced with fresh medium with or without GÖ6976 (1.5 *μ*M), these cells were then cultured under hypoxia or normoxic condition. Pictures were taken at indicated time using a digital camera system coupled to a microscope (Leica, Germany). The width of the scratches was analyzed using ImageJ software (National Institutes of Health, Bethesda, MD), and the migration area at each time point was normalized to its corresponding area at 0 hours.

### 2.9. Statistical Analysis

All data are expressed as mean ± SD. Values of *P* < 0.05 were considered statistically significant. Two treatment groups were compared by using unpaired Student's *t*-test. Multiple group comparisons were done by one-way ANOVA using a least significant difference post hoc analysis. All analyses were performed with SPSS 19.0.

## 3. Results

### 3.1. HDAC4 Is Phosphorylated after Cerebral Ischemia

**Figure 1:**
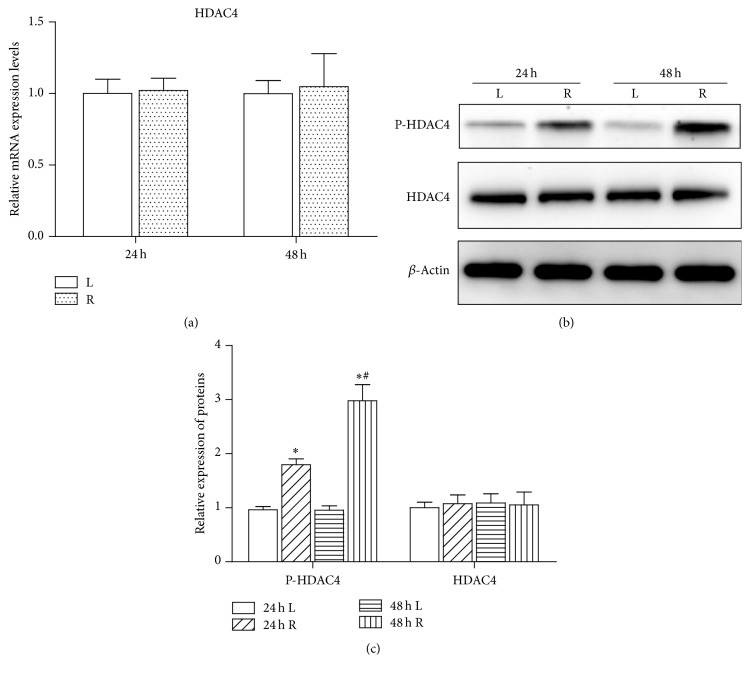
Phosphorylated HDAC4 is upregulated in MCAO rats. (a) Expression changes of HDAC4 in the right ischemic cortex and the left nonischemic cortex were examined by qPCR after 24 hours and 48 hours of reperfusion (*n* = 5/group). ((b) and (c)) Western blot showing the protein levels of phosphorylated HDAC4 in the right ischemic cortex and the left nonischemic cortex at the indicated time points. Quantitative analysis is shown in (c). Data are presented as mean ± SD (^*∗*^*P* < 0.05 versus 24 h L; ^#^*P* < 0.05 versus 48 h L).

To investigate whether HDAC4 is involved in cerebral ischemia, qRT-PCR was performed to evaluate the expression of HDAC4 in rat MCAO model. As shown in Figure 1(a), focal ischemia had no significant effect on the expression of HDAC4 after 24 h and 48 h of reperfusion when compared with the right ischemic cortex and left nonischemic cortex (*P* > 0.05). Since protein phosphorylation has been implicated in the regulation of HDACs activity, we further detected the levels of phosphorylated HDAC4 after cerebral ischemia. Western blot experiments (Figures 1(b) and 1(c)) showed that phosphorylated HDAC4 in the right ischemic cortex was remarkably increased after cerebral ischemia when compared with the left nonischemic cortex (*P* < 0.05).

### 3.2. Phosphorylated HDAC4 Contributes to Angiogenesis In Vivo

**Figure 2:**
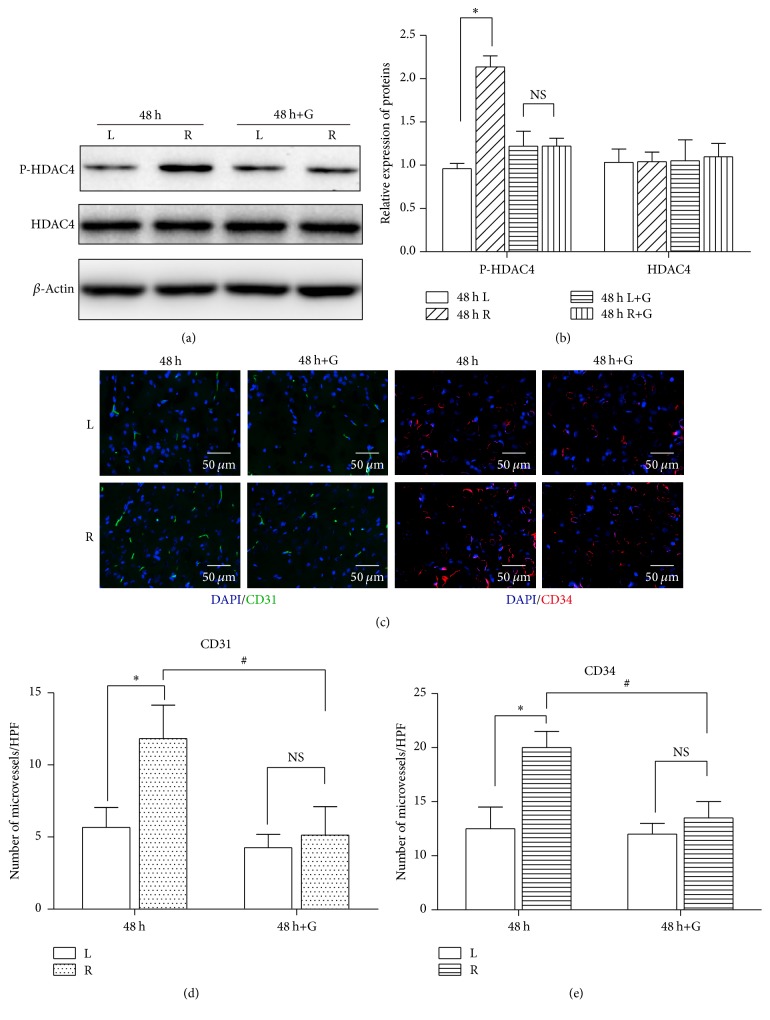
Phosphorylated HDAC4 contributes to angiogenesis in vivo. ((a) and (b)) MCAO rats were injected with GÖ6976 or vehicle control for 48 hours (*n* = 5/group). Expression change of P-HDAC4 in the ischemic penumbra cortex and contralateral cortex was measured by Western blot analysis. Quantitative analysis is shown in (b). ((c), (d), and (e)) Microvessel density was assessed 48 hours after injection of GÖ6976 or vehicle control. Representative images are shown in (c) and the number of microvessels is quantified in (d) and (e). Data are presented as mean ± SD. NS, nonstatistically significant. ^*∗*^*P* < 0.05 versus L; ^#^*P* < 0.05 versus 48 h+G. Scale bar = 50 *μ*m.

To evaluate the effect of phosphorylated HDAC4 in cerebral ischemia, HDAC4 phosphorylation inhibitor GÖ6976 was used in this study. Western blot results showed that rats given GÖ6976 can effectively block the phosphorylation of HDAC4 after 48 h of reperfusion when compared with the vehicle injection group (Figures 2(a) and 2(b)). Most strikingly, accompanied with the suppression of phosphorylated HDAC4, immunofluorescence results indicated that the number of PECAM-1 positive cells or CD34 positive cells in the ischemic penumbra area was significantly decreased in GÖ6976 treated group compared with the vehicle treated group (*P* < 0.05) (Figures 2(c), 2(d), and 2(e)). Those data indicate that inhibition of HDAC4 phosphorylation may suppress the endogenous angiogenesis induced by cerebral ischemia.

### 3.3. Phosphorylated HDAC4 Regulates Angiogenesis In Vitro

**Figure 3:**
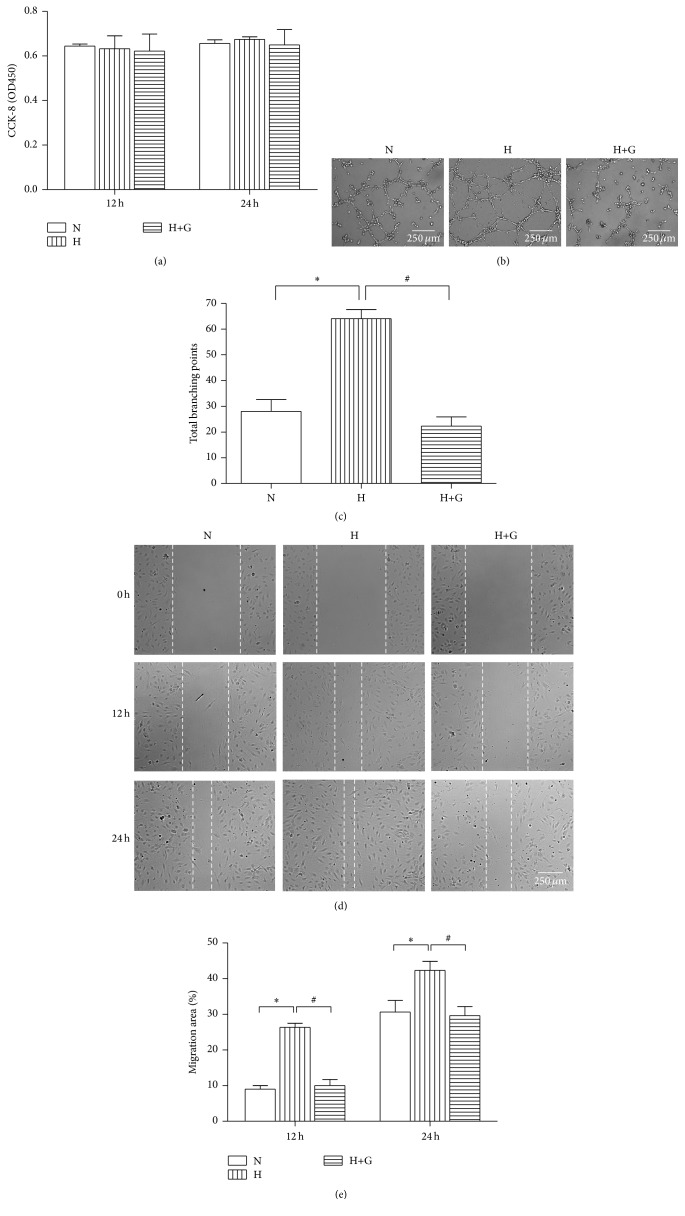
Phosphorylated HDAC4 regulates angiogenesis in vitro. (a) RBMEC cells treated with GÖ6976 or vehicle control were cultured in normoxic condition (21% O_2_, 5% CO_2_) or under hypoxia (1% O_2_, 5% CO_2_, and 94% N_2_) for 12 or 24 hours. CCK-8 cell proliferation analysis was performed at the indicated time. ((b) and (c)) Tube formation assay was assessed 12 hours after treatment with or without GÖ6976. Representative images are shown in (b), and total loops are quantified in (c). ((d) and (e)) Wound scratch assay was analyzed at the indicated time. Representative images are shown in (d), and migration areas are quantified in (e). Data are presented as mean ± SD. N: cells cultured in normoxic condition; H: cells cultured in hypoxia condition. ^*∗*^*P* < 0.05 versus N; ^#^*P* < 0.05 versus H. Scale bar = 250 *μ*m.

**Figure 4:**
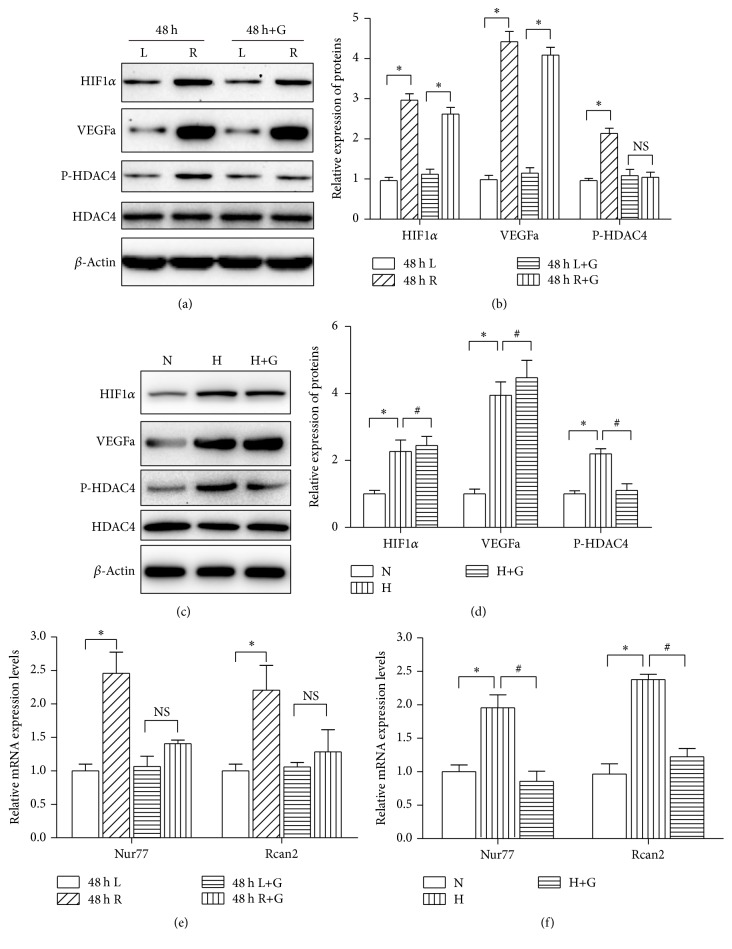
Phosphorylated HDAC4 regulates HIF-VEGF downstream genes expression. ((a) and (b)) Expression change of HIF1*α*, VEGFa, and P-HDAC4 in the ischemic penumbra cortex and contralateral cortex at the indicated time points. Quantitative analysis is shown in (b). ((c) and (d)) Expression change of HIF1*α*, VEGFa, and P-HDAC4 in RBMEC cells cultured in the model of hypoxia. Quantitative analysis is shown in (d). (e) Nur77 and Rcan2 mRNA expression was determined after GÖ6976 or vehicle injection (*n* = 5/group). Data are presented as mean ± SD. NS, nonstatistically significant. ^*∗*^*P* < 0.05 versus L. (f) RBMEC cells treated with GÖ6976 or vehicle control were cultured for 24 hours. The expression of Nur77 and Rcan2 was quantified by qRT-PCR at the indicated time points. Data are presented as mean ± SD. ^*∗*^*P* < 0.05 versus N; ^#^*P* < 0.05 versus H.

We then investigated the effect of phosphorylated HDAC4 on angiogenesis in vitro. We first measured the expression change of HDAC4 in a cell hypoxia model which mimics the pathophysiology of cerebral ischemia. In accordance with our in vivo result, the levels of phosphorylated HDAC4 were significantly increased in endothelial cells exposed to hypoxia (1% O_2_) (Figures 4(c) and 4(d)), indicating that HDAC4 phosphorylation widely occurs during ischemia/hypoxia. Then the impacts of phosphorylated HDAC4 on endothelial cells proliferation, migration, and tube formation were explored in the hypoxia model. CCK-8 cell proliferation analysis showed that HDAC4 phosphorylation or dephosphorylation in endothelial cells had no significant effects on cell proliferation (Figure 3(a)). Scratch wound assay indicated that phosphorylated HDAC4 enhanced the motility of endothelial cells, while suppressing HDAC4 phosphorylation reduced the migration capabilities of endothelial cells compared to the normoxia and hypoxia group (Figures 3(b) and 3(c)). To determine their effects on tube formation, the cells were seeded on Matrigel and cultured for 12 hours. After culturing on Growth Factor Reduced Matrigel, endothelial cells shortly stimulated with hypoxia had better capillary-like structures and more numbers of tube branches, while blocking phosphorylated HDAC4 in endothelial cells with GÖ6976 significantly inhibited their tube formation capacity, as demonstrated by less number of tube branches (Figures 3(d) and 3(e)). Collectively, our in vivo and in vitro results indicate that HDAC4 is functioned in a phosphorylated way and plays an important role in neovascularization induced by cerebral ischemia.

### 3.4. Phosphorylated HDAC4 Mediates HIF-VEGF Signaling Induced Vessel Formation

HIF-VEGF signaling is an important signaling pathway in the regulation of angiogenesis after ischemic stroke. As shown in Figures 4(a) and 4(b), we found that the protein levels of HIF1*α* and VEGFa were increased in the ischemic penumbra after ischemia stroke. The expressions of HIF1*α* and VEGFa were also significantly upregulated after endothelial cells were exposed to hypoxia (Figures 4(c) and 4(d)). To address whether phosphorylated HDAC4 mediated HIF-VEGF signaling induced angiogenesis, qRT-PCR was performed to examine the genes expression of HIF-VEGF downstream genes in MCAO rats. Nur77 and Rcan2 are angiogenesis related genes which are controlled by Class II HDACs [18, 19] and also transcribe activation by VEGF [20, 21]. As shown in Figure 4(e), the expressions of Nur77 and RCAN2 were induced approximately 2.5-fold and 2-fold in the MCAO vehicle injection group (*P* < 0.05), while suppression of HDAC4 phosphorylation in the MCAO GÖ6976 injection group decreased the magnitude of induction of Nur77 and RCAN2 expression (*P* > 0.05) and attenuated angiogenesis induced by HIF-VEGF signaling (Figures 2(c), 2(d), and 2(e)). We further tested the impact of phosphorylated HDAC4 on genes expression in endothelial cells in the context of HIF-VEGF activation. As we expected, the expressions of Nur77 and RCAN2 were upregulated (approximately 2-fold and 2.5-fold) after 24-hour hypoxia when compared with the normoxic group. Blocking the phosphorylation of HDAC4 with GÖ6976 inhibited RCAN2 and Nur77 expression compared with the vehicle control (*P* < 0.05) (Figure 4(f)) and suppressed HIF-VEGF signaling mediated tube formation and cell migration (Figures 3(b), 3(c), 3(d), and 3(e)). These data suggest that phosphorylated HDAC4 serves as a molecular switch for HIF-VGEF signaling and regulates HIF-VEGF signaling mediated angiogenesis.

## 4. Discussion

Acetylation or deacetylation of chromatin proteins and transcription factors is part of a complex signaling system that is largely involved in the control of gene expression following neurological disorders [22]. Accumulating evidences have implicated HDACs in the progression of neuronal ischemic injury [23] and have shown that HDACs exert protective effects in attenuating neuronal injury after cerebral ischemia [24]. Thereby, identification of the specific roles of HDACs may provide clues for novel therapy of stroke. The results of this study have identified the mechanisms by which HDAC4 serves as an important HIF-VEGF signal-sensitive molecule to modulate angiogenesis responses to cerebral ischemia.

Most of the ischemic strokes in human occur in the area of middle cerebral artery [25, 26]; transient middle cerebral artery occlusion (tMCAO) in rodents is more clinically relevant compared to other models and is widely utilized in experimental stroke studies on focal cerebral ischemia [27, 28]. Previous studies have used tMCAO model to study angiogenesis after ischemia stroke [29–31]. In this study, we use rat tMCAO model and found that the expression of HDAC4 was unaltered after cerebral ischemia, but phosphorylation of HDAC4 was significantly upregulated in MCAO rats. Consistently, this phenomenon was also confirmed in a mouse MCAO model which showed that cerebral ischemia had no significant effect on HDAC4 mRNA levels [32]. Previous studies have demonstrated that dephosphorylation of HDAC4 caused nuclear accumulation of HDAC4 in neurons and led to ataxia telangiectasia neurodegeneration [33]. Intracellular trafficking of HDAC4 promotes neuronal apoptosis and represses the transcriptional activity of survival factors in neurons [34]. Phosphorylated HDAC4 nuclear export increases fetal cardiac genes expression and plays a dominant role in the regulation of cardiac hypertrophy and heart failure [35]. Our data along with published studies suggest that phosphorylated HDAC4 may play a functional role in cerebral ischemia.

HDACs are reported to be regulators of vascular and convincing evidences in vitro which have revealed that phosphorylation of HDACs plays an important role in VEGF induced angiogenesis [18, 36, 37]. We detected the phosphorylation of HDAC4 in hypoxia models which mimic the pathophysiology of stroke in vitro. Interestingly, the phenomenon of HDAC4 phosphorylation was also observed in endothelial cells stimulated with hypoxia. Previous studies have demonstrated that the PKC/PKD pathway is required for HDAC4 phosphorylation [38–40] and phosphorylation of HDAC4 in response to VEGF was abolished by the PKC/PKD inhibitor GÖ6976 [18]. Then, we used GÖ6976 to study the function of phosphorylated HDAC4. In the present study, our Western blotting results demonstrated that endothelial cells treated with GÖ6976 or MCAO rats intraperitoneally injected with GÖ6976 can effectively suppress the phosphorylation of HDAC4. Furthermore, in vivo immunofluorescence studies indicated that MCAO rats given GÖ6976 markedly attenuated endogenous angiogenesis after cerebral ischemia. Blocking hypoxia induced HDAC4 phosphorylation with GÖ6976 resulted in suppression of endothelial cells tube formation and cell migration in vitro, essential steps and functions responsible for angiogenesis. Taken together, these results demonstrate that phosphorylated HDAC4 may play a functional role in angiogenesis after cerebral ischemia.

Induction of angiogenesis after ischemic stroke stimulates endogenous recovery mechanisms, which promote neurogenesis, facilitate synaptogenesis, increase neuronal and synaptic plasticity, and therefore in turn improve the neurological outcome [41]. Among the factors capable of modulating angiogenesis characterized to date, HIF-VEGF signaling is the most studied and is the main mechanism that controls angiogenesis after stroke due to the pathophysiology of cerebral ischemia/hypoxia [29, 42]. Recent advance in HDACs demonstrated that HDAC4 phosphorylation and dephosphorylation, which shuttles between the cytoplasm and the nucleus, are signal responsive regulators and consequently modulate signaling pathway functions [38, 43–45]. To name a few, HDAC4 is phosphorylated by AMPK signaling and regulated metabolism associated transcription factors and genes expression in the liver [46]. Considering the fact that HDAC4 is phosphorylated in response to signals and functions as a signaling regulator, we speculate that phosphorylated HDAC4 may be HIF-VEGF signaling responsive and exert its function through HIF-VEGF signaling. In support of this hypothesis, we found that the HIF-VEGF signal was activated in ischemic regions after cerebral ischemia, and the phosphorylated HDAC4 protein was remarkably increased after the activation of HIF-VEGF signaling. In vitro study results also indicated that the levels of phosphorylated HDAC4 increased in the context of activated HIF-VEGF signaling. Rcan2 and Nur77 are among the early VEGF response genes implicated in angiogenesis [20, 21] and are also controlled by Class II HDACs [18]. Therefore, Rcan2 and Nur77 were selected to investigate the function of HDAC4 in HIF-VEGF signal pathway. Treatment with GÖ6976 suppressed the expression of Nur77 and RCAN2 both in vivo and in vitro. These results suggest that HDAC4 phosphorylation activates HIF-VEGF signaling and thereby mediates HIF-VEGF signaling induced endogenous angiogenesis in peri-infarct cortex after cerebral ischemia.

## 5. Conclusion

In summary, our in vivo and in vitro studies demonstrated that HDAC4 is phosphorylated after cerebral ischemia. Phosphorylation of HDAC4 may contribute to the angiogenesis in the ischemic brain through a mechanism involving HIF-VEGF signaling. It demonstrates a role for phosphorylated HDAC4 mediated HIF-VEGF signal and regulated HIF-VEGF downstream genes expression in cerebral ischemia, which may provide novel therapeutic target for cerebral ischemia.
